# Toward a transcriptomic framework for ultrasound neuromodulation: A perspective on gene expression and regional brain sensitivity

**DOI:** 10.1162/IMAG.a.1294

**Published:** 2026-07-08

**Authors:** Joline M. Fan, Ehsan Tadayon, Andrew D. Krystal, Keith R. Murphy

**Affiliations:** Department of Neurology, University of California, San Francisco, CA, United States; Weill Institute for Neurosciences, University of California, San Francisco, CA, United States; Department of Psychiatry and Behavioral Sciences, University of California, San Francisco, CA, United States; Attune Neurosciences, San Francisco, CA, United States

**Keywords:** low-intensity focused ultrasound, transcranial ultrasound stimulation, ultrasound parameters, genetic mechanisms

## Abstract

Non-invasive transcranial ultrasound stimulation (TUS) enables deep brain therapeutic exploration at unprecedented scale. However, its optimal use is limited by the uncertainty surrounding ultrasound sensitivity across brain regions and cell types. This uncertainty often forces selection of sub-optimal parameter–target treatment paradigms guided solely by precedent, rather than an unbiased search of the larger combinatorial space. In principle, human brain-wide gene expression data could allow for a refinement of the search space based on known mechanisms and associated gene expression. In this perspective, we discuss the practicality of genetically informed TUS parameter search in humans using the Allen Brain Atlas and incorporating a broad set of genes related to the hypothesized mechanisms of TUS neuromodulation. We define principal component expression patterns across the brain, enabling dimensionality reduction and spatial clustering of ultrasound-relevant gene expression data. We identify regional clusters of covarying gene expression profiles across the brain topology that are likely to have similar responsivity to TUS. These findings may explain previous perplexities around highly variant neuronal response across brain areas and highlight the need to optimize stimulation parameters in the context of brain region and its molecular profile.

## 1 Introduction

Transcranial ultrasound stimulation (TUS) has rapidly emerged as a versatile scientific and therapeutic tool for non-invasively modulating neural circuits within the human brain (Legon & Strohman, 2024; Murphy et al., 2025). Unlike current non-invasive methods, TUS offers the ability to modulate cortical and subcortical brain targets with millimeter precision (Martin et al., 2025; Phipps et al., 2024). However, the process of translating TUS to different brain targets and networks suffers from the variant ultrasound sensitivity and effects across brain regions and cell types (Gao et al., 2025; Murphy et al., 2024; Yaakub et al., 2023; Yu et al., 2024). Prior experimental studies have demonstrated that mechanosensitive ion channels, for example, play a causal role in mediating ultrasound responsiveness. As such, genetic knock-down or overexpression has been shown to confer different ultrasound sensitivities, supporting a model by which differential gene expression across the cortex would yield differences in ultrasound parameterization. To fully optimize this process would require an enormous TUS parameter search across brain regions, which is particularly challenged by the relatively long duration effects of TUS even with a short stimulation paradigm. As a result, many clinical trials have largely relied on precedent parameter selection, raising the risk of suboptimal outcomes. Further guidance on how to approach parameterization across the brain is greatly needed to help with the identification of optimal stimulation paradigms. Here we explore the utility of human brain-wide gene expression data to identify subdivisions of differential ultrasound sensitivities within the brain.

## 2 Diverse Genetic Mechanisms Underlying Ultrasound Neuromodulation

At present, we gain many of our intuitions from DBS, but there are stark differences in mechanisms. DBS creates electrical gradients that directly alter cellular membrane potentials, while TUS engages biochemical sensors of thermal effects or mechanical force that indirectly alter neural activity (Blackmore et al., 2019; Dell’Italia et al., 2022; Kamimura et al., 2020). Furthermore, sequential interactions between biophysical effects, the mechanosensitive ion channels, and downstream signaling cascades further complicate the multidimensional response in TUS. Previous efforts have used the transcriptomic patterns to predict vulnerability to polygenic, brain network diseases (Grothe et al., 2018; Hartl et al., 2021). These approaches inspire potential application for the creation of a mechanistic framework of modeling TUS sensitivity across the brain. In principle, human brain-wide gene expression data could allow for a refinement of the search space based on known mechanisms and associated gene expression.

Significant research has attributed genetic components to the direct biophysical sensing of TUS and subsequent transduction into membrane voltage changes (Chalfie, 2009; He et al., 2025). For instance, lipid-mediated signaling occurs as membrane distortion disrupts lipid rafts in the membrane, releasing GM1 substrates for interaction with membrane-tethered enzymes such as phospholipase D (PLD2) (Petersen et al., 2016, 2019). The resulting substrates can then interact with various mechanosensitive ion channels such as PIEZO1, TRPA1, and TREK-1, to give rise to a change in membrane potential (Brohawn, Su, et al., 2014; Kubanek et al., 2016; Montell, 2005; Oh et al., 2019; Petersen et al., 2019; Prieto et al., 2018; S. P. Wang et al., 2016; Yoo et al., 2022; Zhu et al., 2023). Transmembrane proteins, such as integrins, act as mechanosensors linking the extracellular matrix to the intracellular signaling pathways, potentially engaging integrin-linked kinase pathways and cytoskeletal remodeling (Katsumi et al., 2004; Ross et al., 2013).

## 3 Spatial Transcriptomic Analysis of Ultrasound-Relevant Genes

Leveraging the current understanding of TUS mechanisms, we performed a spatial gene expression analysis using publicly available data from the Allen Institute Human Brain Atlas (Hawrylycz et al., 2012) and ultrasound-related genes. A list of 56 ultrasound-relevant genes across 12 mechanosensitive gene families was compiled through systematic literature review, spanning mechanosensitive ion channels, voltage-gated channels, gap junctions, integrins, lipid signaling mediators, and caveolins (Table 1; Supplementary Figs. S1 and S2). We then extracted stable spatial expression profiles (n = 46 genes) (Markello et al., 2021), applied dimensionality reduction via principal components analysis (10 components capturing 90.6% variance; Supplementary Fig. S3), and performed unsupervised clustering to identify co-expression networks that grouped brain regions by shared genetic mechanosensitive gene expression, which should confer dynamic ultrasound responsiveness. Spatially distinct ultrasound-related gene expression may reflect cell-type differences and differential mechanical specialization across the brain. Intriguingly, hierarchical clustering of spatial gene expression patterns did not reveal grouping by gene class (Monte Carlo permutation test, p = 0.12, Supplementary Fig. S4), suggesting that genes with similar functions are not spatially co-expressed, perhaps reflecting functional redundancy. PCA identified dominant spatial axes of gene expression variability (Supplementary Fig. S5): PC1 separates cortex from subcortical structures, PC2 distinguished insular and anterior cingulate regions from occipital cortex, and PC3 clearly delineates the thalamus, highlighting that predominant gene variability aligned with neuroanatomical organization.

**Table 1.** Molecular mechanisms implicated in TUS neuromodulation.

Category	Genes identified (n = 56)*	Mechanism of action
**Piezo Channels**	**PIEZO1**, **PIEZO2**	Primary mechanosensors directly activated by membrane stretch; facilitate cation influx and rapid depolarization; confer ultrasound sensitivity of neurons and endothelial cells (Prieto et al., 2018; S. P. Wang et al., 2016; Zhu et al., 2023)
**TRP Channels**	**TRPV1**, **TRPV2**, **TRPV3**, TRPV4, TRPA1, **PKD1**, **PKD2**, **TRPC1**, **TRPC3**, **TRPC4**, **TRPC5**, TRPC6, **TRPM2**, **TRPM3**, **TRPM4**, **TRPM7**, TRPM8	Respond to mechanical stretch, temperature, osmotic pressure, chemical, and lipid signaling (Christensen & Corey, 2007; Eijkelkamp et al., 2013; Lin & Corey, 2005; Montell, 2005; Oh et al., 2019); mediate ultrasound-induced calcium influx in neurons and astrocytes (Christensen & Corey, 2007; Eijkelkamp et al., 2013; Lin & Corey, 2005; Montell, 2005; Oh et al., 2019)
**K2P Channels**	**KCNK1**, **KCNK2**, **KCNK3**, KCNK4, **KCNK5**, **KCNK9**, **KCNK10**, KCNK18	Background “leak” potassium channels modulated by membrane stretch; regulate resting membrane potential and neuronal excitability (Brohawn, Campbell, et al., 2014; Brohawn, Su, et al., 2014; Kubanek et al., 2016)
**ASIC**	**ASIC1**, **ASIC2**, **ASIC3**, **ASIC4**	Proton-gated channels with demonstrated mechanosensitive properties, particularly in sensory neurons (Chen & Wong, 2013; Cheng et al., 2018; Ruan et al., 2021)
**Voltage-Gated Na+ (Nav)**	**SCN1A**, **SCN2A**, SCN5A, **SCN8A**, SCN11A	Mechanosensitive gating properties; conductance influenced by membrane deformation and altered voltage gradients (Beyder et al., 2010; Israel et al., 2019; Kubanek et al., 2016; Morris & Juranka, 2007)
**T-type Ca2+ (Cav3)**	**CACNA1G**, **CACNA1H**, **CACNA1I**	Mechanical modulation of low-threshold calcium channels contributing to neuronal firing and burst activity (Shin et al., 2003; R. Wang & Lewin, 2011)
**Inward Rectifier K+ (Kir)**	**KCNJ10**, **KCNJ2**	Mechanosensitive inward rectifier potassium channels involved in glial potassium buffering and neuronal excitability (Hoger et al., 2002; Sancho et al., 2022)
**Connexin/Pannexin**	**GJA1**, **GJB6**, **PANX1**, **PANX2**	Gap junction and hemichannel proteins modulated by mechanical stress; mediate intercellular communication and ATP release (Cherian et al., 2005; Grimston et al., 2008; Yang et al., 2022)
**Chloride/Anion Channels**	**BEST1**, **LRRC8A**	Volume-regulated anion channels responding to cell swelling and mechanical stress (Fischmeister & Hartzell, 2005)
**Integrins**	**ITGB1**, **ITGB2**, ITGB3, **ITGB8**	ECM-cytoskeleton mechanotransducers at focal adhesions; transduce mechanical forces to intracellular signaling pathways (Jaudon & Cingolani, 2024; Katsumi et al., 2004; Sun et al., 2016)
**Phospholipase D**	**PLD1**, **PLD2**	Membrane tension sensors; enzymatic signaling cascades resulting from mechanical deformation of lipid rafts (Petersen et al., 2016, 2019)
**Caveolin**	**CAV1**, **CAV2**, CAV3	Caveolae scaffolding proteins forming mechanosensitive membrane domains that sense and transduce mechanical forces (Echarri & Del Pozo, 2015; Sinha et al., 2011)

* Bolded genes represent those that were available in the Allen Human Brain Atlas, passed the stability threshold (Abagen toolbox, ibf_threshold = 0.4) (Arnatkevičiūtė et al., 2019; Hawrylycz et al., 2012; Markello et al., 2021), and included in the gene clustering analysis.

**Fig. 1.**
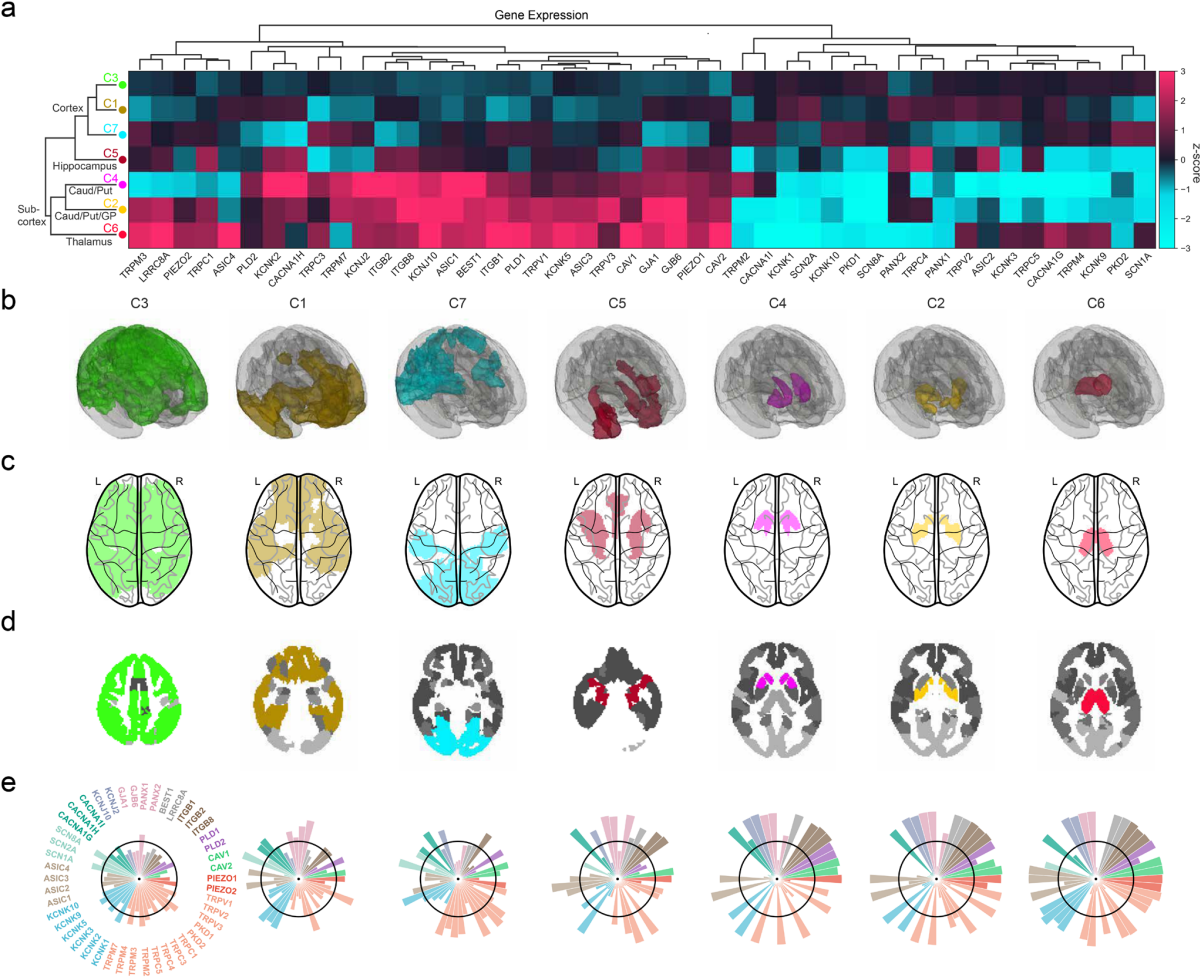
Spatial clustering and gene family expression profiles of TUS-relevant genes. (a) Heatmap showing mean z-scored expression of 46 mechanosensitive genes across K = 7 clusters (silhouette = 0.257). Genes (columns) are ordered by hierarchical clustering to reveal co-expression patterns; clusters (rows) are ordered by hierarchical similarity. Row dendrogram and left color bar indicate K = 7 parent cluster membership (pink = high expression, cyan = low expression). (b) Three-dimensional brain renderings with each cluster highlighted in its assigned color against a translucent gray brain surface. Columns correspond to clusters C1–C7. (c) Glass brain projections in axial view showing the spatial extent of each cluster. (d) Axial slices at the z-coordinate of maximum cluster extent; target cluster in color, other clusters in grayscale. (e) Bidirectional radar plots showing mean z-scored expression patterns for 12 gene families (Piezo, TRP, K2P, ASIC, Nav, Cav, Kir, Connexin, Chloride, Integrin, Phospholipase, Caveolin). Black circle = z-score of 0; vertices outside = elevated expression; vertices inside = reduced. Cluster composition: C1 (n = 71, cortical), C2 (n = 10, subcortical), C3 (n = 158, cortical), C4 (n = 8, subcortical), C5 (n = 17, mixed), C6 (n = 8, subcortical), C7 (n = 60, cortical). Caud, caudate; GP, globus pallidus; Put, putamen.

Gene clustering analysis recapitulated the most prominent anatomic division occurring between cortical and subcortical structures (Fig. 1a). To ensure robust cluster selection, K-means clustering was repeated across 100 random initializations with n_init = 10 (Supplementary Fig. S6), and silhouette scores were averaged with 95% confidence intervals computed. This robust silhouette analysis identified K = 5 as optimal (mean silhouette = 0.249) for broad parcellation, with K = 7 representing a local maximum (mean silhouette = 0.257; Fig. 1) and K = 13 representing a local minima providing finer granularity (Supplementary Fig. S7). At K = 5, dominant clusters separated medial prefrontal/temporal cortices from dorsolateral frontal/parietal regions with distinct subcortical clusters. At K = 7, additional sub-clusters emerged, providing finer spatial resolution of cortical and subcortical structures (Fig. 1b–d). Monte Carlo validation (10,000 random 46-gene sets) revealed moderate similarity between TUS gene clusters and random gene clusters (mean ARI = 0.45 at K = 5, 0.40 at K = 7, permuted across 10,000 random seeds), indicating that while global gene expression partially drives clustering, TUS genes contribute additional specificity beyond random gene sets (Supplementary Fig. S8). These findings demonstrate spatially organized clusters of expression similarity (Fig. 1a), suggesting that these clusters may share ultrasound sensitivity. In this framework, protocol response may be inferred from the previous examination of other brain areas within the same spatial transcriptomic cluster.

We next examined the constituent expression profiles of 12 mechanosensitive gene families across the K = 7 clustering solution (Fig. 1e). Radar plots revealed distinct transcriptional fingerprints differentiating cortical from subcortical clusters. The three subcortical clusters (C2, C4, C6) exhibited markedly elevated expression of Piezo1 channels, inwardly rectifying potassium channels (Kir), integrins, and chloride channels, while showing substantially reduced voltage-gated sodium channel (Nav) expression. In contrast, the three cortical clusters (C1, C3, C7) displayed more balanced expression profiles across gene families. Notably, cluster C6, representing the thalamus, showed the highest Piezo family expression (z = 1.85) alongside elevated ASIC (z = 1.91), chloride (z = 2.71), and caveolin (z = 2.95), suggesting this subcortical region may exhibit enhanced direct mechanosensitivity to ultrasound stimulation. The Nav family showed the most pronounced cortical–subcortical differentiation, with cortical clusters showing mildly elevated expression (C3 z = 0.34, C7 z = 0.32), while subcortical clusters C2 and C4 showed marked reductions (z = −2.29 and z = −2.16, respectively). These gene family profiles provide a framework for understanding how regional differences in mechanosensitive channel expression may contribute to differential TUS responsivity across brain structures.

## 4 The Quest for Parameter Space Optimization

The search for optimal TUS parameters has been largely empiric, relying on trial-and-error approaches and precedent across an expansive, multidimensional parameter space. Within this high-dimensional space, our transcriptomic clustering analysis further emphasizes the added complexity of regional genetic variability impacting TUS sensitivity and responsiveness. Indeed, prior work in animals has demonstrated that optimal parameters derived from grid optimization in one brain region may not translate to other parts of the brain (Gao et al., 2025; Murphy et al., 2024; Yaakub et al., 2023; Yu et al., 2024). Although the Allen Atlas Human Brain Atlas is based on a small number of cadavers, the resulting clusters may explain perplexities in the field such as the lack of GABAergic modulation in the dACC despite strong changes in the adjacent PCC, given the distinct clustering of the ACC and PCC (Supplementary Fig. S7) (Yaakub et al., 2023). It may also explain differences in the optimal stimulation protocols that yield excitatory or inhibitory responses across arousal centers (Murphy et al., 2024) or in the magnitude of responses of ultrasound evoked potentials between motor cortex and hippocampus (Tseng et al., 2021), which represent distinct clusters.

This framework may further explain differences in the time courses of behavioral effects across regions, including analgesic effects (Kim et al., 2024) or other unexpected behavioral findings, including the therapeutic effects observation with focused ultrasound of the anterior thalamus, which is commonly associated with worsening mood in epilepsy using deep brain electrical stimulation (Fan et al., 2024). Yet, another example is the finding that the BNST is relatively TUS insensitive, despite high expression of Piezo1 (Murphy et al., 2024; Zhu et al., 2023). This heterogeneity likely has important implications, including the expected neural response, dose curve, and durability of effect, given the range of mechanisms at play that may be involved in acute versus sustained or neuroplastic responses. However, stimulation to regions within the same cluster or clusters with similar genetic profiles may lead to similar magnitudes of region-specific network response (Folloni et al., 2019), and a potential consideration is that within-cluster brain regions may then benefit from the similar TUS protocols.

## 5 Future Directions

This perspective sets the stage for a biologically informed approach to TUS dosing and parameterization by leveraging transcriptomic profiles to predict regional sensitivity. By integrating the Allen Brain Atlas gene expression data with systematic literature-derived mechanosensitive gene sets, we identified spatially organized clusters of co-varying gene expression that likely reflect underlying differences in ultrasound responsiveness. However, these differences remain theoretical and beckon further examination. In order to test differential sensitivities, a common neuromodulation outcome must be established that is agnostic to circuit function. Furthermore, methods for maintaining focal pattern integrity across regions must also be established to control for physical strain and pressure differences. Controlling these conditions may be set through use of organoid culture matched with transcription profiles of gene clusters. In vivo studies that minimize circuit confounds may include systemic stimulation across brain regions and neurochemical signal readouts at the focus of stimulation. Finally, extending this approach to individual patient-derived transcriptomic data presents a direct path toward individual, biologically informed TUS targeting parameter selection and dosing strategy, moving the community toward a long-term goal of precision neuromodulation tailored to regional biology.

## 6 Materials and Methods

### 6.1 Gene selection through systematic PubMed literature search

Candidate genes were selected through a six-step procedure (Supplementary Fig. S1). First, 12 mechanosensitive gene families were identified from independent reviews spanning 3 tiers: direct TUS mechanosensors (Piezo, TRP, K2P, ASIC) (Prieto & Maduke, 2024; Ranade et al., 2015; Song et al., 2023), broader mechanotransduction channels (Nav, Cav, Kir, Connexin, Chloride, Integrin) (Di et al., 2023; Heppenstall & Lewin, 2006; Legon & Strohman, 2024; Li et al., 2024), and force-from-lipid mediators (Phospholipase, Caveolin) (Head et al., 2014; Petersen et al., 2016). Second, all human members of these families were enumerated via HGNC nomenclature, yielding 147 candidate genes. Third, PubMed literature validation was performed using systematic search queries across 14 mechanosensitivity categories (108 queries, 10,893 articles screened) to confirm each gene’s relevance to neural mechanosensation. Fourth, an inclusion threshold of 10 or more supporting articles was applied, retaining 59 genes. Fifth, three genes were excluded based on expert review (PLD3 for lacking phospholipase D enzymatic activity; SCN9A for peripheral-only expression; CACNA1C as an L-type rather than T-type calcium channel), yielding 56 genes. Sixth, genes were filtered for availability in the Allen Human Brain Atlas, reducing the final set to 46 genes across 12 families (Table 1).

### 6.2 Preprocessing

Gene expression data for all available ultrasound-related genes were extracted from the Allen Human Brain Atlas. Processing and normalization of expression profiles were performed using the *abagen* toolbox (https://abagen.readthedocs.io/; Arnatkevičiūtė et al., 2019; Hawrylycz et al., 2012; Markello et al., 2021), which aligns tissue samples to a standard cortical and subcortical parcellation, filters low-quality probes, and averages expression across donors. Ibf_threshold was set to 0.4. For cortical regions, we used a 300-region functional parcellation in MNI space, and for subcortical regions, we used a 32-region parcellation, yielding a total of 332 regions of interest (ROIs). The *abagen* pipeline was configured with the following parameters: ibf_threshold=0.4, lr_mirror=“bidirectional”, and missing= “interpolate”. See full Abagen report in Supplementary Materials.

### 6.3 Dimensionality reduction and clustering

To reduce dimensionality while preserving dominant spatial variance, principal components analysis (PCA) was applied to the processed expression matrix. The top 10 components captured 90.6% of the total variance (Supplementary Fig. S3). PCA loading terms were extracted to quantify each gene’s contribution to the principal components. K-means clustering was performed on the PCA-reduced data to identify spatial clusters of gene expression across brain regions. To address sensitivity to random initialization, we employed robust silhouette analysis: for each K value (2–14), K-means was run with 10 initializations and repeated across 100 random seeds, with mean silhouette scores and 95% confidence intervals computed. This identified K = 5 as optimal for K ≥ 3 (excluding the trivial cortex/subcortex split at K = 2) and K = 7 as a local maximum, justifying dual-resolution analysis (Supplementary Fig. S6). Monte Carlo validation compared TUS gene clusters against 10,000 random 46-gene sets using Adjusted Rand Index (ARI), revealing moderate architecture-driven similarity with TUS-specific contributions. Clusters and genes were organized by agglomerative hierarchical clustering (metric=Euclidean, method=“average”) and visualized via dendrogram-ordered heatmap.

To examine the spatial correspondence between gene expression-derived clusters and canonical functional brain networks (Thomas Yeo et al., 2011), we performed an overlap analysis using volumetric brain atlases in MNI152 space. Both the gene cluster image and Yeo 7-network parcellation were resampled to match the MNI152 space and nearest neighbor interpolation. Each gene cluster was compared with each Yeo network mask, and the overlapping voxels were computed for each combination of cluster and resting-state network label.

### 6.4 Monte Carlo simulation

Monte Carlo simulation (n = 10,000 iterations) assessed cluster specificity relative to general brain transcriptomic organization. For each iteration, 46 genes were randomly selected from the full expression matrix (15,633 genes) and processed identically to TUS genes: PCA dimensionality reduction (10 components) followed by K-means clustering with silhouette-based optimization (K = 3–15). Adjusted Rand Index (ARI) quantified similarity between TUS gene cluster assignments and random gene set cluster assignments. Silhouette scores, cumulative variance explained, and optimal cluster number were compared between TUS genes and the null distribution. Statistical significance was determined by percentile ranking within the null distribution.

Gene expression similarity was assessed by hierarchical clustering (average linkage, Euclidean distance) of K = 7 cluster-level mean z-scored expression vectors, producing a dendrogram leaf ordering of the 46 genes. To test whether genes from the same family are spatially co-expressed (i.e., positioned adjacently in this ordering), we computed the number of adjacent gene pairs sharing the same family label and compared it with a null distribution generated by randomly permuting family labels across genes 10,000 times. The one-sided p-value was calculated as the proportion of permutations yielding an equal or greater number of adjacent same-family pairs than the observed value. The test statistic (z-score) was computed as (observed − null mean)/null SD.

## Supplementary Material

Supplementary Material

## Data Availability

Code is available at https://github.com/jolinefan/TUSgeneClustering.git
