# Supplementary material for: Toward a transcriptomic framework for ultrasound neuromodulation: A perspective on gene expression and regional brain sensitivity
Source: Imaging Neurosci (Camb). 2026 Jul 8;4:IMAG.a.1294. doi: 10.1162/IMAG.a.1294 (PMC13347599; doi:10.1162/IMAG.a.1294)
Supplement: Supplementary Material [file IMAG.a.1294_supp.pdf]

SUPPLEMENTARY METHODS

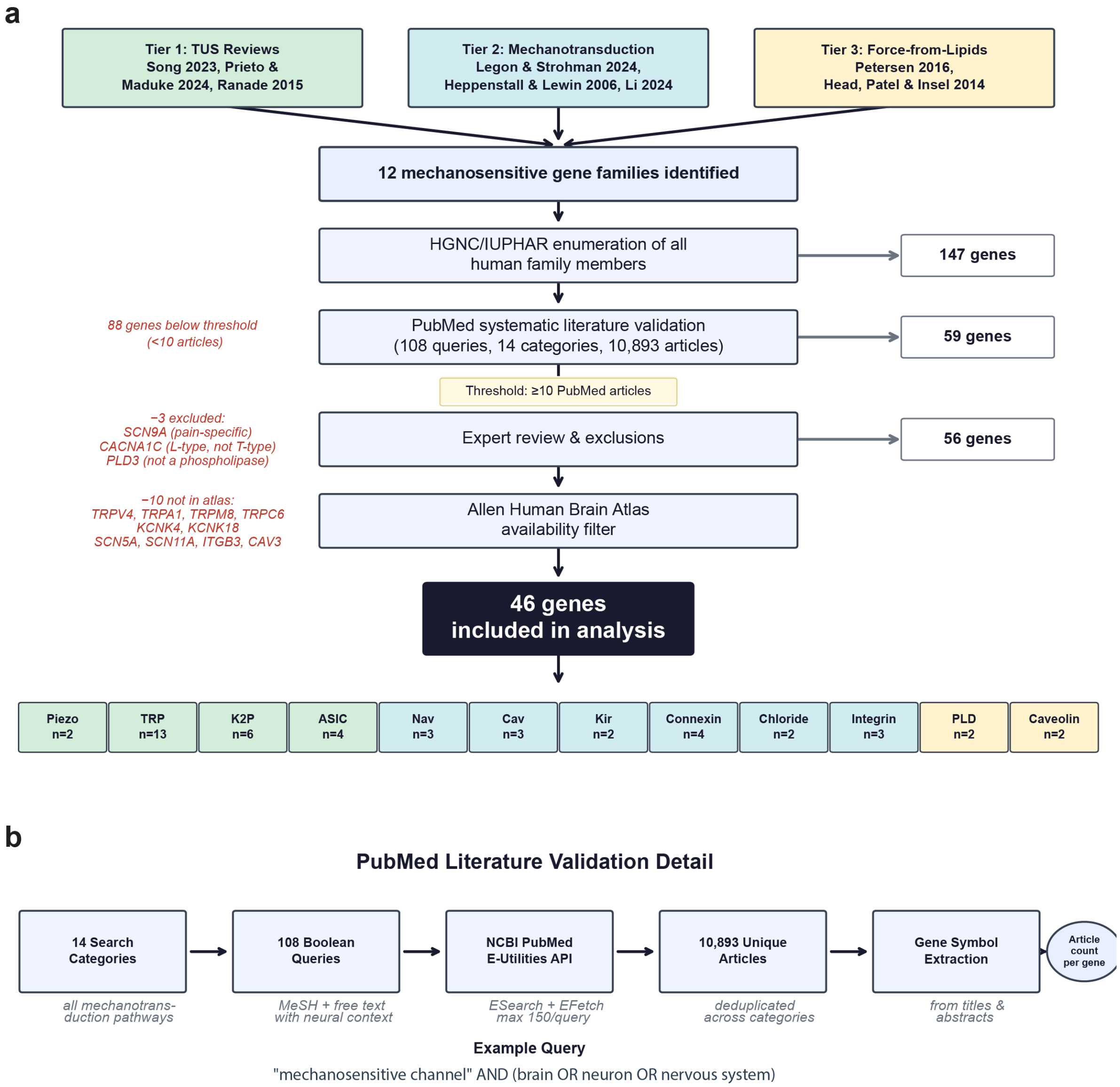

Figure S1. Systematic gene selection workflow for mechanosensitive TUS gene candidates. (a) Flowchart illustrating the stepwise gene selection methodology. Twelve mechanosensitive gene families were identified from three tiers of independent review literature: Tier 1, TUS-specific reviews (Song et al., 2023; Prieto & Maduke, 2024; Ranade et al., 2015); Tier 2, broader mechanotransduction reviews (Legon & Strohman, 2024; Heppenstall & Lewin, 2006; Li et al., 2024); Tier 3, force-from-lipids mechanotransduction (Petersen et al., 2016; Head, Patel & Insel, 2014). All human gene members were enumerated via HGNC/IUPHAR nomenclature (147 genes). Genes were filtered by PubMed systematic literature validation (threshold ≥10 articles; 59 genes remaining), expert exclusions with documented rationale (3 genes removed: SCN9A, CACNA1C, PLD3; 56 genes remaining), and Allen Human Brain Atlas microarray availability (10 genes absent; 46 genes retained for spatial clustering analysis). Family-level gene counts are shown at bottom, color-coded by review tier. (b) PubMed literature validation methodology. 108 Boolean search queries spanning 14 mechanotransduction pathway

categories were executed against the NCBI PubMed E-Utilities API (ESearch + EFetch). Results were deduplicated across categories, yielding 10,893 unique articles from which gene symbols were extracted from titles and abstracts to generate per-gene article counts.

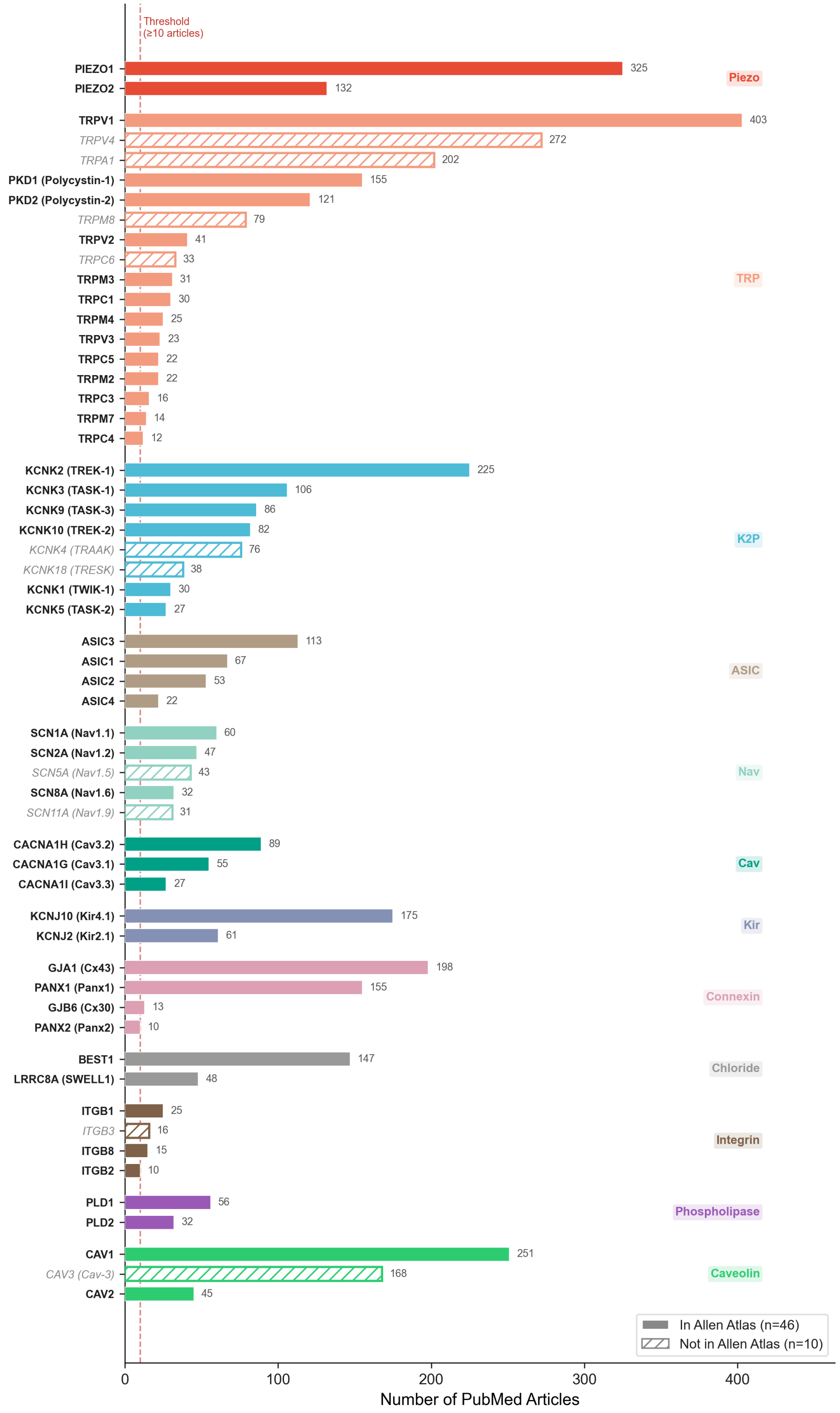

Figure S2. PubMed systematic literature validation of mechanosensitive gene candidates and their article counts. Article counts for all 56 genes that passed the PubMed literature threshold (≥10 articles), organized by gene family. Each row represents a gene; bar chart shows the total number of unique PubMed articles mentioning each gene across all categories. Solid

bars denote the 46 genes available in the Allen Human Brain Atlas and included in the spatial clustering analysis (gene names in bold); hatched bars denote the 10 genes that passed literature validation but were absent from the atlas microarray platform (gene names in italic). Colored brackets (left) and bar colors indicate gene family membership. Genes are ordered by family and by descending article count within each family.

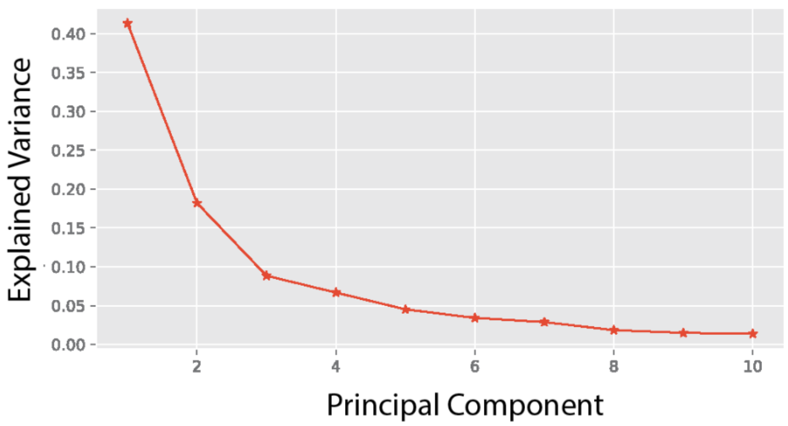

Figure S3. **Methods related to dimensionality reduction via principal components analysis.** Plot reveals explained variance per principal component. Across 10 principal components, 90.6% of variance is explained in the spatial gene expression data.

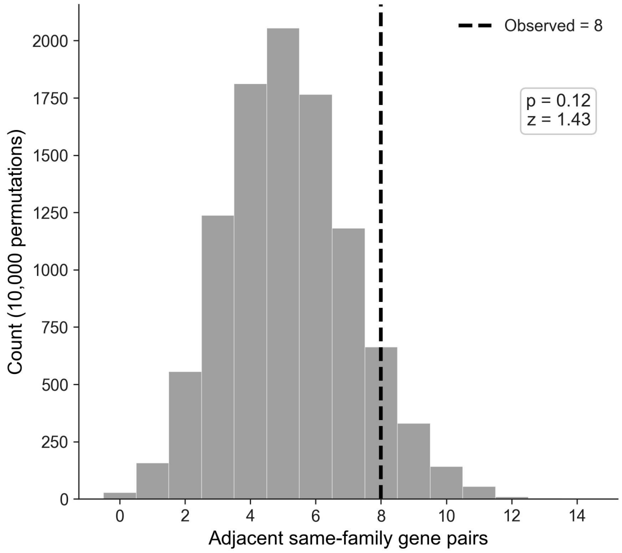

Figure S4: Gene Family Clustering Along the Expression Dendrogram (a) Permutation test assessing whether genes from the same family cluster together along the hierarchical dendrogram axis (Figure 1a). The metric is the number of adjacent gene pairs sharing the same family in the dendrogram leaf order. The observed value (8 adjacent same-family pairs, dashed line) is compared against a null distribution generated by randomly permuting family labels across the 46 genes 10,000 times (gray histogram). The observed clustering does not significantly exceed chance ($p = 0.12$, $z = 1.43$), indicating that the expression-based gene ordering does not recapitulate gene family membership.

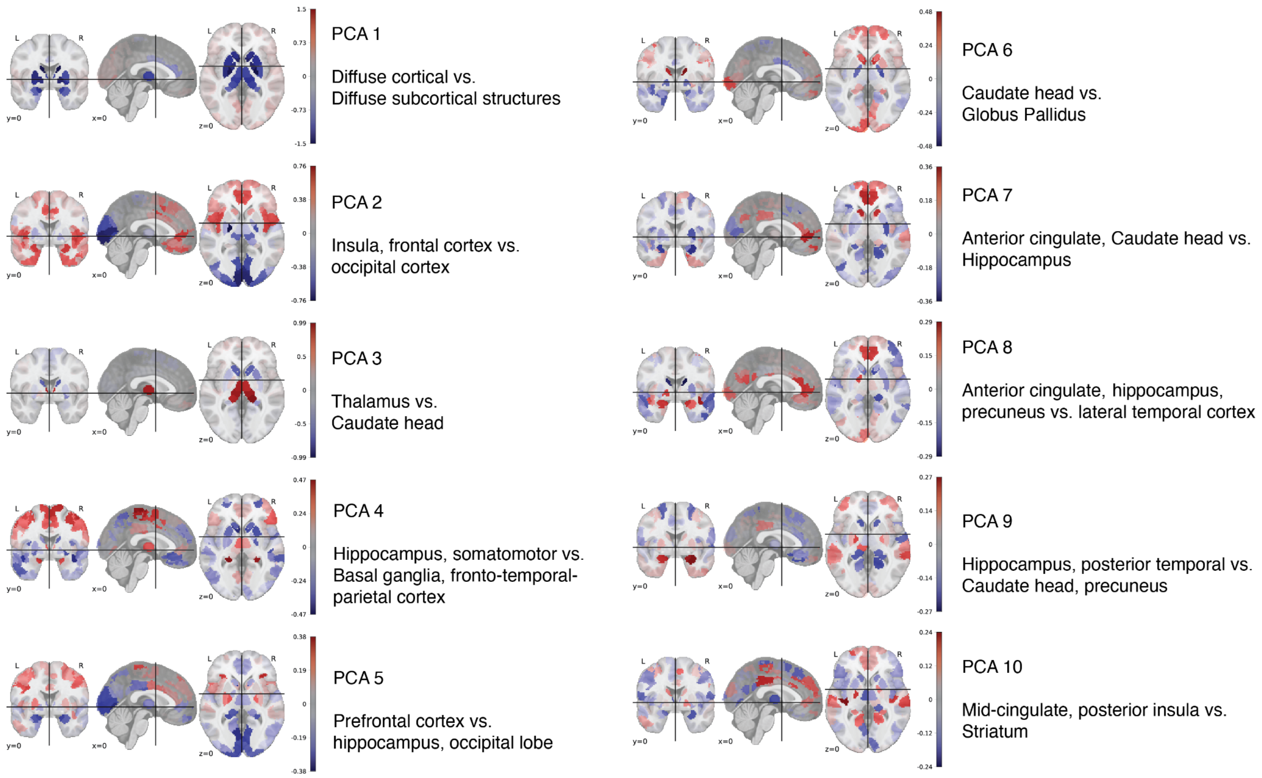

Figure S5. Principal components of spatial gene expression profiles. Spatial maps of all ten principal components (PC1–PC10) derived from 46 mechanosensitive gene expression values across 332 brain regions, ordered by decreasing explained variance. Together, these components capture 90.6% of total spatial variance in the gene expression matrix. Each component is rendered on coronal, saggital, and axial brain slices with red indicating regions with positive loadings and blue indicating negative loadings. PC1 captures the dominant cortical–subcortical expression gradient, reflecting the primary axis of transcriptomic variation. Subsequent components delineate finer neuroanatomical distinctions.

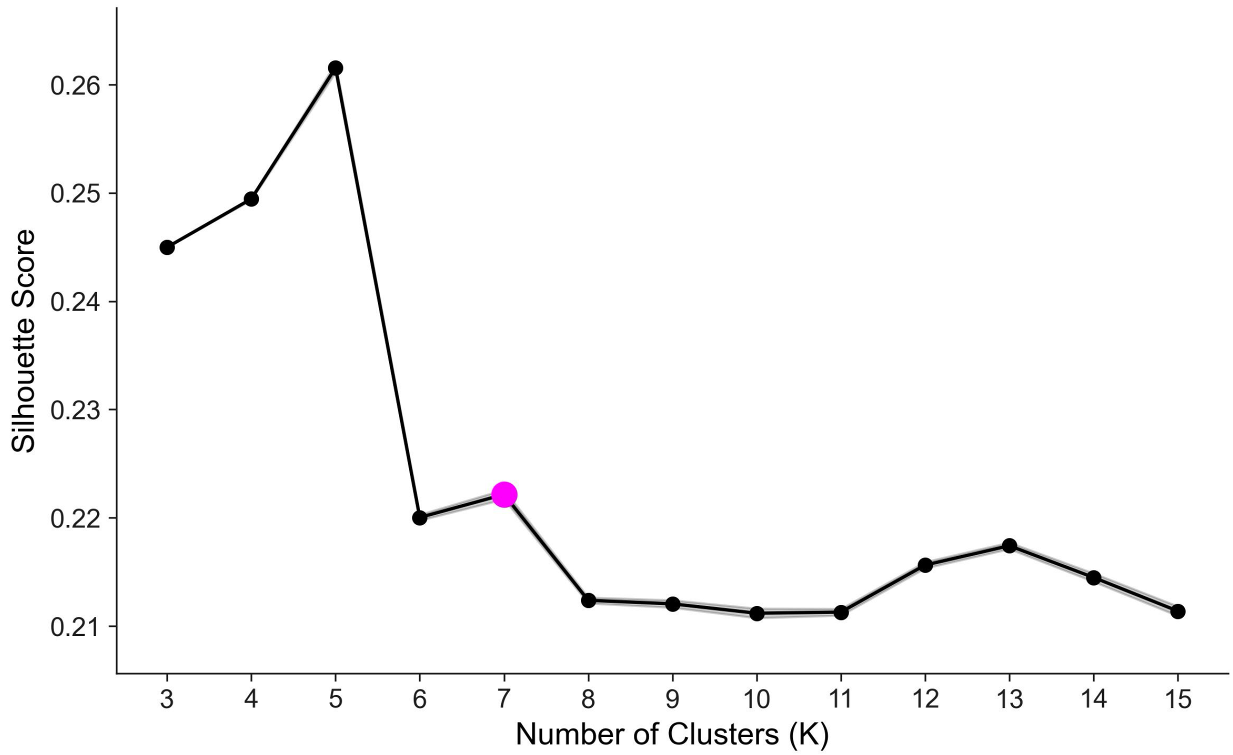

Figure S6. Robust silhouette analysis for cluster number selection. Mean silhouette scores (black line) with 95% confidence intervals (shaded region) across K=3 to K=15 clusters, computed over 100 random initializations with n_init=10. K=2 was excluded as it trivially separates cortical from subcortical regions. K=5 (silhouette = 0.249) was selected as the optimal parcellation. K=7 (magenta circle; silhouette = 0.257) is a local maximum selected for the primary analysis to capture additional regional heterogeneity. The decline in silhouette scores beyond K=7 suggests diminishing cluster coherence with increasing granularity.

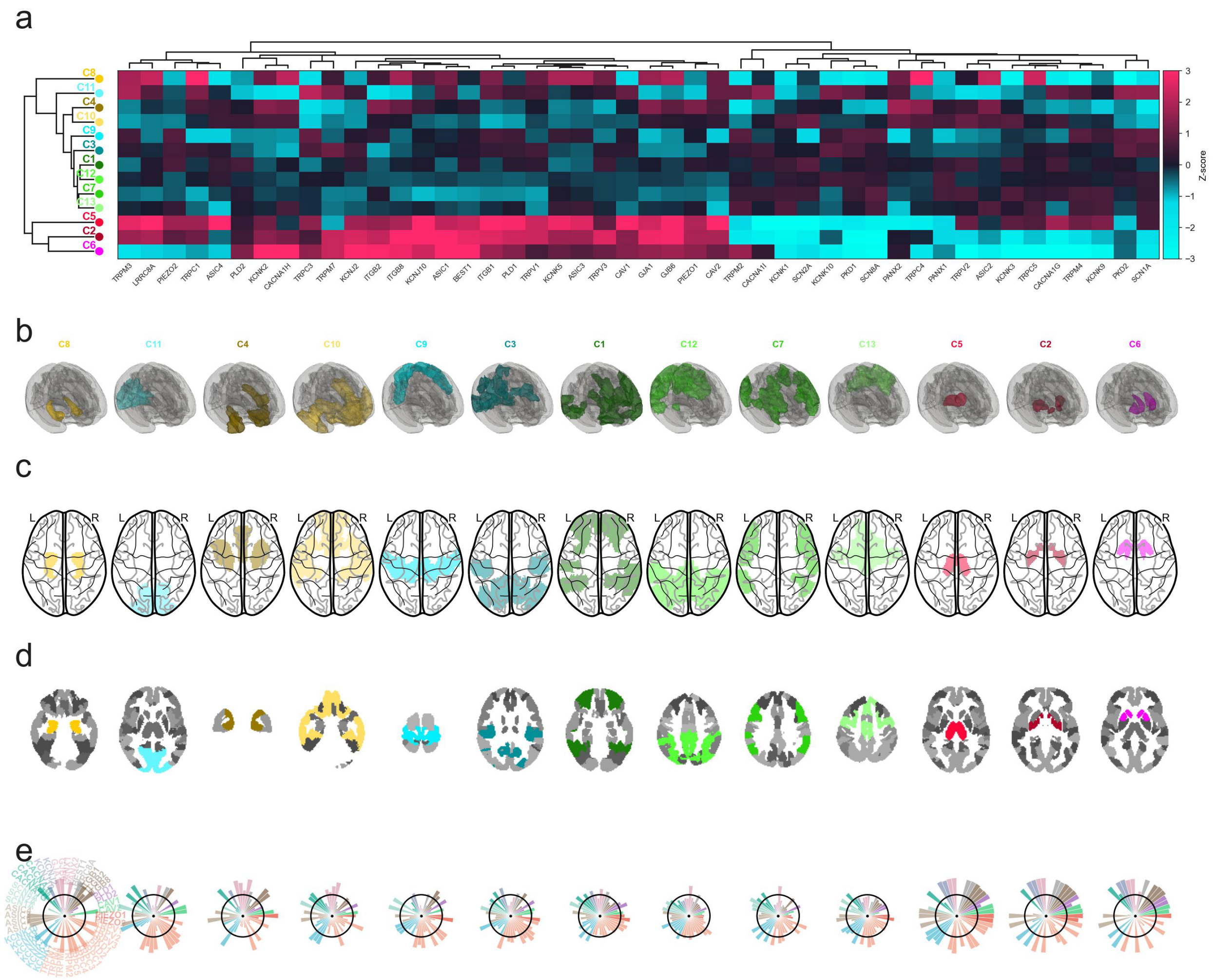

Figure S7. Spatial clustering and gene family expression profiles of TUS-relevant genes at a finer resolution (k=13). (a) Heatmap showing mean z-scored expression of 46 mechanosensitive genes across K=13 clusters as in main Fig 1. (b) Three-dimensional brain renderings with each cluster highlighted in its assigned color against a translucent gray brain surface. Columns correspond to clusters C1–C7. (c) Glass brain projections in axial view showing the spatial extent of each cluster. (d) Axial slices at the z-coordinate of maximum cluster extent; target cluster in color, other clusters in grayscale. (e) Bidirectional radar plots showing mean z-scored expression patterns for twelve gene families (Piezo, TRP, K2P, ASIC, Nav, Cav, Kir, Connexin, Chloride, Integrin, Phospholipase, Caveolin). Black circle = z-score of 0; vertices outside = elevated expression; vertices inside = reduced.

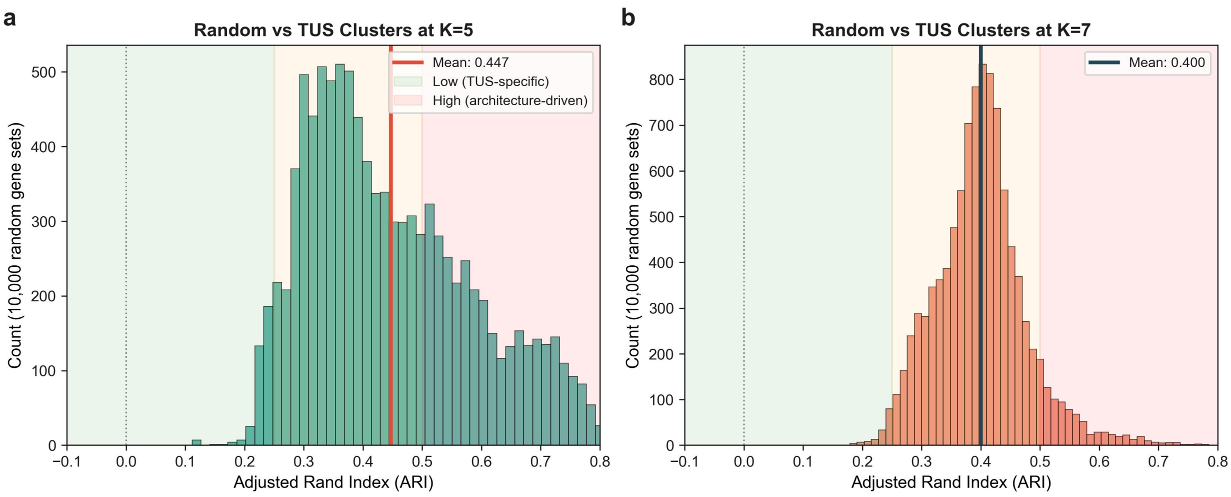

Figure S8. Monte Carlo validation of TUS gene clustering specificity. (a) Distribution of Adjusted Rand Index (ARI) values comparing TUS gene cluster assignments to 10,000 random 46-gene sets clustered at K=5 (optimal). Red line indicates mean ARI (0.45). Green shading = low similarity (TUS-specific); orange = moderate; red = high (architecture-driven). (b) ARI distribution at K=7 (granular). Dark line indicates mean ARI (0.40).

SUPPLEMENTAL METHODS

Abagen Full Report

Regional microarray expression data were obtained from 6 post-mortem brains (1 female, ages 24.0–57.0, mean ± SD: 42.50 ± 13.38) provided by the Allen Human Brain Atlas (https://human.brain-map.org). Data were processed with the *abagen* toolbox (https://github.com/rmarkello/abagen) using a 332-region volumetric atlas in MNI space.

Probe Filtering and Selection

First, microarray probes were reannotated using data provided by [A2019N]; probes not matched to a valid Entrez ID were discarded. Next, probes were filtered based on their expression intensity relative to background noise [Q2002N], such that probes with intensity less than the background in ≥50.00% of samples across donors were discarded.

When multiple probes indexed the expression of the same gene, we selected and used the probe with the most consistent pattern of regional variation across donors (i.e., differential stability) calculated as follows:

$$\Delta_s(p) = [1 / C(N, 2)] \sum_{i=1}^{N-1} \sum_{j=i+1}^{N} \rho[B_i(p), B_j(p)]$$

where ρ is Spearman's rank correlation of the expression of a single probe, *p*, across regions in two donors B_i and B_j, and *N* is the total number of donors. Here, regions correspond to the structural designations provided in the ontology from the AHBA.

Tissue Sample Assignment

The MNI coordinates of tissue samples were updated to those generated via nonlinear registration using the Advanced Normalization Tools (ANTs; https://github.com/chrisfilo/alleninf). Samples were assigned to brain regions in the provided atlas if their MNI coordinates were within 2 mm of a given parcel.

To reduce the potential for misassignment, sample-to-region matching was constrained by hemisphere and gross structural divisions (i.e., cortex, subcortex/brainstem, and cerebellum), such that, for example, a sample in the left cortex could only be assigned to an atlas parcel in the left cortex. All tissue samples not assigned to a brain region in the provided atlas were discarded.

Normalization

Inter-subject variation was addressed by normalizing tissue sample expression values across genes using a robust sigmoid function [F2013J]:

$$x\_norm = 1 / (1 + \exp(-(x - \langle x \rangle) / IQR_x))$$

where $\langle x \rangle$ is the median and IQR_x is the normalized interquartile range of the expression of a single tissue sample across genes.

Normalized expression values were then rescaled to the unit interval:

$$x\_scaled = (x\_norm - \min(x\_norm)) / (\max(x\_norm) - \min(x\_norm))$$

Gene expression values were then normalized across tissue samples using an identical procedure. Samples assigned to the same brain region were averaged separately for each donor and then across donors, yielding a regional expression matrix.

***References*:**

[A2019N]: Arnatkevičiūtė, A., Fulcher, B. D., & Fornito, A. (2019). A practical guide to linking brain-wide gene expression and neuroimaging data. *Neuroimage*, 189, 353-367.

[F2013J]: Fulcher, B. D., Little, M. A., & Jones, N. S. (2013). Highly comparative time-series analysis: the empirical structure of time series and their methods. *Journal of the Royal Society Interface*, 10(83), 20130048.

[Q2002N]: Quackenbush, J. (2002). Microarray data normalization and transformation. *Nature Genetics*, 32(4), 496-501.
